# Supplementary material for: Molecular Analysis of the Complete Genome of a Simian Foamy Virus Infecting Hylobates pileatus (pileated gibbon) Reveals Ancient Co-Evolution with Lesser Apes
Source: Viruses. 2019 Jul 3;11(7):605. doi: 10.3390/v11070605 (PMC6669568; doi:10.3390/v11070605)
Supplement: Supplementary file 1 [file viruses-11-00605-s001.pdf]

Molecular Analysis of the Complete Genome of a Simian Foamy Virus Infecting *Hylobates pileatus* (pileated gibbon) Reveals Ancient Co-evolution with Lesser Apes

Anupama Shankar[1], Samuel D. Sibley[2], Tony L. Goldberg[2] and William M. Switzer[4,*]

Supplementary Figure. Phylogenetic analysis of foamy virus (FV) envelope (*env*) sequences of 48 taxa using maximum likelihood analysis with FastTree [1]. **A.** Analysis of the complete *env* (3,110 nucleotide positions); **B.** Analysis of 2,186 nucleotide positions without the receptor binding domain (RBD) of the surface (SU) protein region; **C.** Analysis of the RBD of SU region of *env* (810 nucleotide positions). **Old World apes:** Simian foamy virus (SFV) pvr, P*an troglodytes verus* (chimpanzee), GenBank accession number U04327; SFVptr, *Pan troglodytes* (chimpanzee), GenBank accession numbers provided in figure; SFVpsc, *Pan troglodytes schweinfurthii* (chimpanzee), Y07725; SFVppy, *Pongo pygmaeus* (orangutan), AJ544579; SFVggo, *Gorilla* (gorilla), NC_039029; other SFVggo GenBank numbers provided in figure; SFVhpi_SAM106, *Hylobates pileatus*, (pileated gibbon), M621235. **Old World monkeys:** SFVcae, *Cercopithecus aethiops* (African green monkey), M74895; SFVmcy, *Macaca cyclopsis* (Formosan macaque), X54482; GenBank accession numbers for other OWM SFV provided in figure (MMU, *Macaca mullata*). **New World monkeys:** SFVcja, *Callithrix jacchus* (common marmoset), GU356395; SFVsxa, *Sapajus xanthosternos* (capuchin), KP143760; SFVaxx, *Ateles* species (spider monkey), EU010385; SFVssc, *Saimiri sciureus* (squirrel monkey), GU356394. **Prosimian (Pro):** SFVocr, *Otolemur crassicaudatus* (brown greater galago), KM233624.
Non-simian mammals (NSM): Equine FV (EFV)eca, *Equus caballus*, AF201902; Bovine FV (BFV)bta, *Bos taurus*, U94514; and Feline FV (FFV)fca, *Felis catus*, Y08851). All trees were rooted with the NSM. Sequence names designated with “hu” were isolated from zoonotically-infected humans. Taxa in green and orange text represent the SFV with RBD identified as clade I and II, respectively [2, 3]. Confidence values for the branching patterns were assessed with the Shimodaira–Hasegawa (S-H) test and are given as probabilities to the left of each branching node. S-H values < 0.7 are not shown. The unit for the scale bar is the number of nucleotide substitutions per nucleotide site.

A.

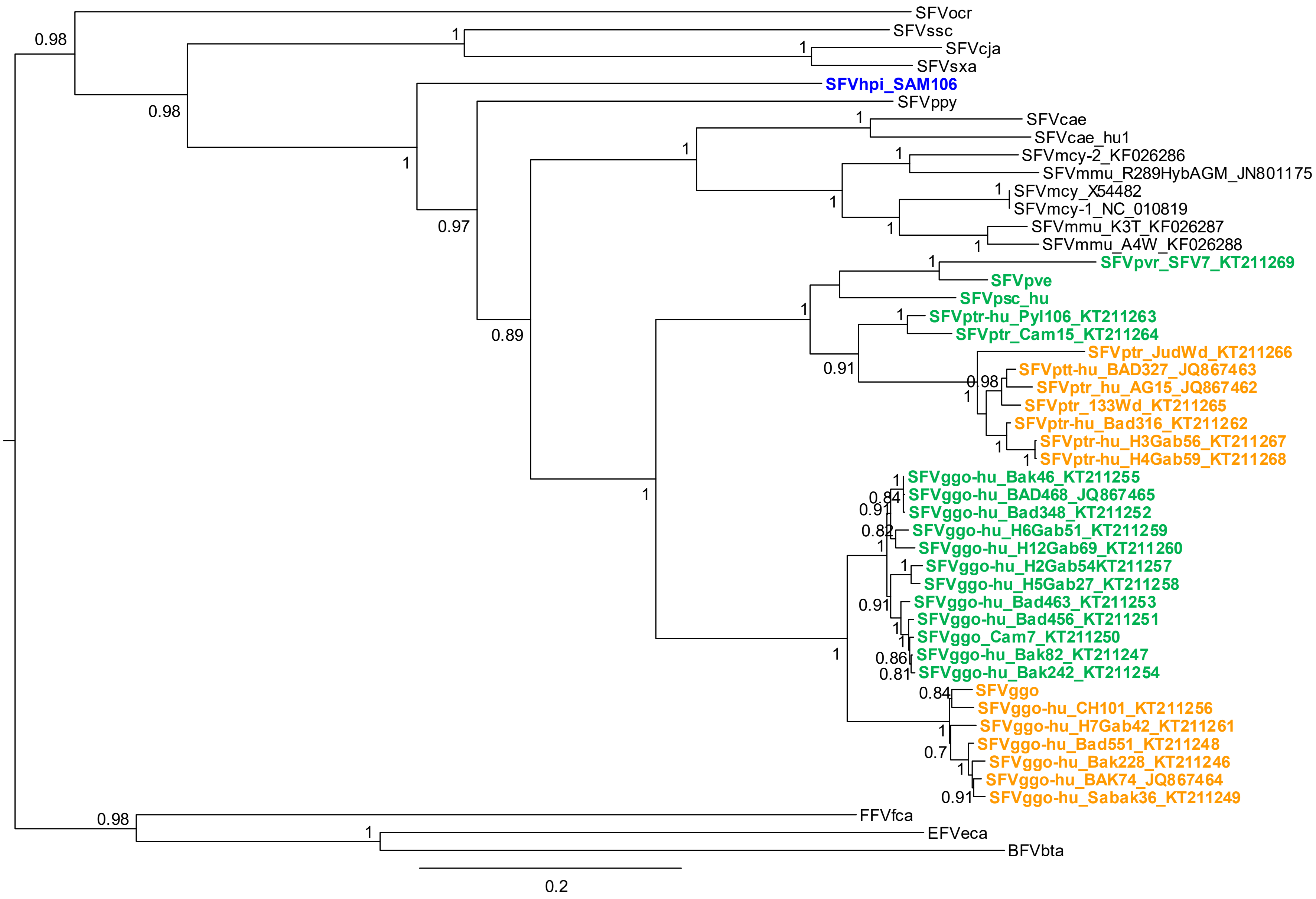
SFVocr
SFVssc
SFVcja
SFVsxa
SFVhpi_SAM106
SFVppy
SFVcae
SFVcae_hu1
SFVmcy-2_KF026286
SFVmmu_R289HybAGM_JN801175
SFVmcy_X54482
SFVmcy-1_NC_010819
SFVmmu_K3T_KF026287
SFVmmu_A4W_KF026288
SFVpvr_SFV7_KT211269
SFVpve
SFVpsc_hu
SFVptr-hu_Pyl106_KT211263
SFVptr_Cam15_KT211264
SFVptr_JudWd_KT211266
SFVptt-hu_BAD327_JQ867463
SFVptr_hu_AG15_JQ867462
SFVptr_133Wd_KT211265
SFVptr-hu_Bad316_KT211262
SFVptr-hu_H3Gab56_KT211267
SFVptr-hu_H4Gab59_KT211268
SFVggo-hu_Bak46_KT211255
SFVggo-hu_BAD468_JQ867465
SFVggo-hu_Bad348_KT211252
SFVggo-hu_H6Gab51_KT211259
SFVggo-hu_H12Gab69_KT211260
SFVggo-hu_H2Gab54KT211257
SFVggo-hu_H5Gab27_KT211258
SFVggo-hu_Bad463_KT211253
SFVggo-hu_Bad456_KT211251
SFVggo_Cam7_KT211250
SFVggo-hu_Bak82_KT211247
SFVggo-hu_Bak242_KT211254
SFVggo
SFVggo-hu_CH101_KT211256
SFVggo-hu_H7Gab42_KT211261
SFVggo-hu_Bad551_KT211248
SFVggo-hu_Bak228_KT211246
SFVggo-hu_BAK74_JQ867464
SFVggo-hu_Sabak36_KT211249
FFVfca
EFVeca
BFVbta
0.2

B.

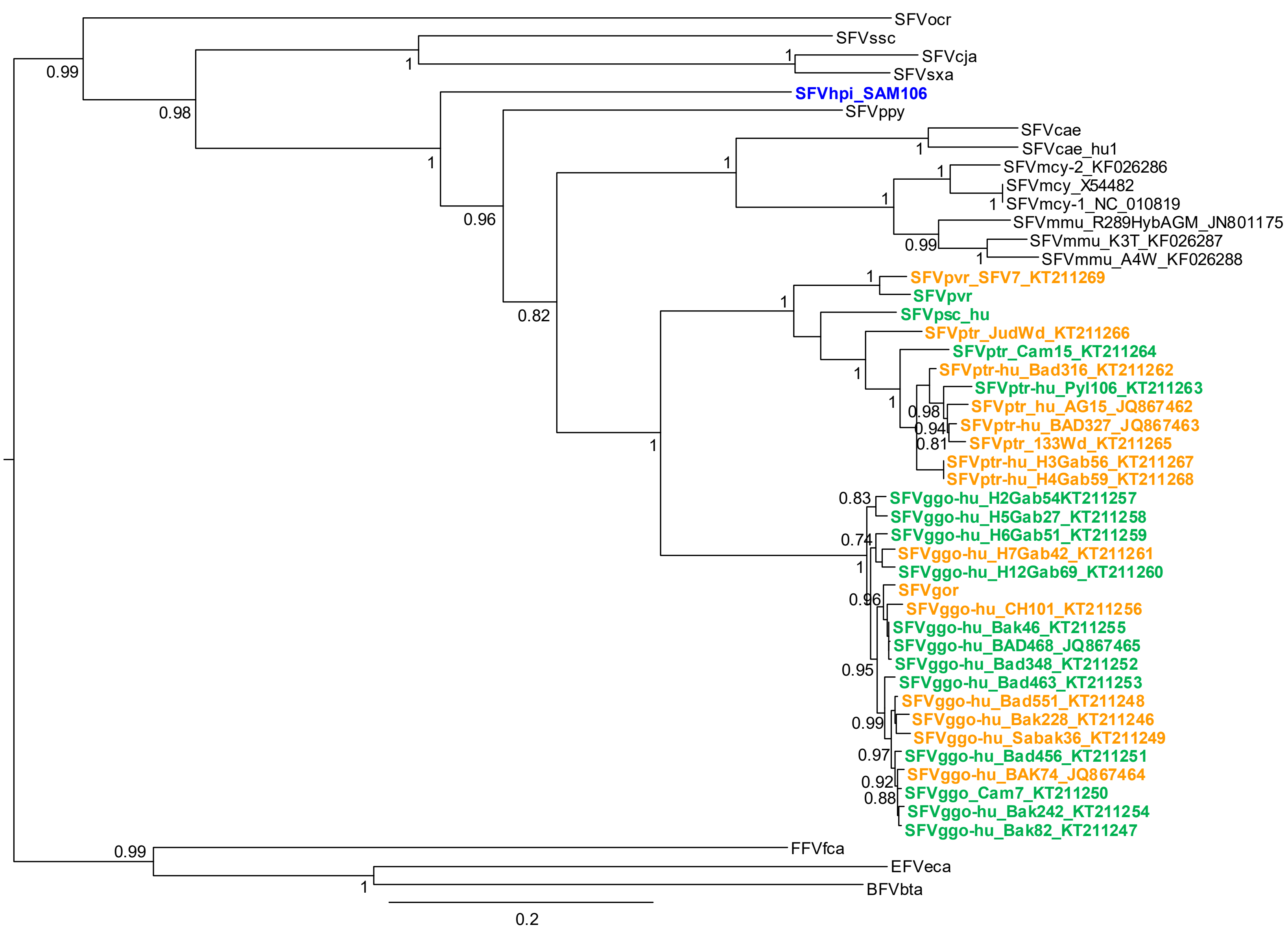
SFVocr
SFVssc
SFVcja
SFVsxa
SFVhpi_SAM106
SFVppy
SFVcae
SFVcae_hu1
SFVmcy-2_KF026286
SFVmcy_X54482
SFVmcy-1_NC_010819
SFVmmu_R289HybAGM_JN801175
SFVmmu_K3T_KF026287
SFVmmu_A4W_KF026288
SFVpvr_SFV7_KT211269
SFVpvr
SFVpsc_hu
SFVptr_JudWd_KT211266
SFVptr_Cam15_KT211264
SFVptr-hu_Bad316_KT211262
SFVptr-hu_Pyl106_KT211263
SFVptr_hu_AG15_JQ867462
SFVptr-hu_BAD327_JQ867463
SFVptr_133Wd_KT211265
SFVptr-hu_H3Gab56_KT211267
SFVptr-hu_H4Gab59_KT211268
SFVggo-hu_H2Gab54KT211257
SFVggo-hu_H5Gab27_KT211258
SFVggo-hu_H6Gab51_KT211259
SFVggo-hu_H7Gab42_KT211261
SFVggo-hu_H12Gab69_KT211260
SFVgor
SFVggo-hu_CH101_KT211256
SFVggo-hu_Bak46_KT211255
SFVggo-hu_BAD468_JQ867465
SFVggo-hu_Bad348_KT211252
SFVggo-hu_Bad463_KT211253
SFVggo-hu_Bad551_KT211248
SFVggo-hu_Bak228_KT211246
SFVggo-hu_Sabak36_KT211249
SFVggo-hu_Bad456_KT211251
SFVggo-hu_BAK74_JQ867464
SFVggo_Cam7_KT211250
SFVggo-hu_Bak242_KT211254
SFVggo-hu_Bak82_KT211247
FFVfca
EFVeca
BFVbta
0.99
0.98
0.96
0.82
0.83
0.74
0.95
0.97
0.92
0.88
0.2

C.

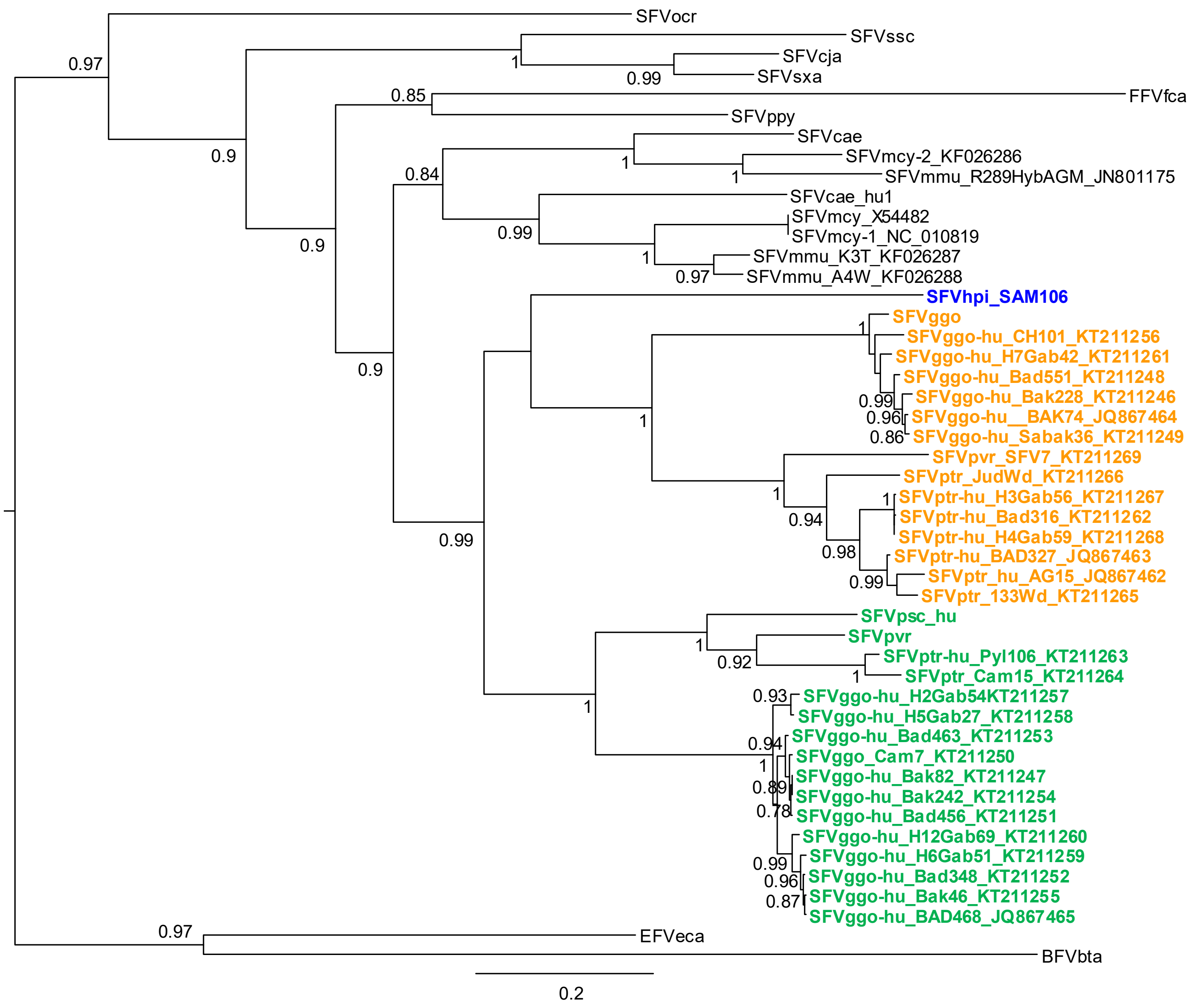
SFVocr
SFVssc
SFVcja
SFVsxa
FFVfca
SFVppy
SFVcae
SFVmcy-2_KF026286
SFVmmu_R289HybAGM_JN801175
SFVcae_hu1
SFVmcy_X54482
SFVmcy-1_NC_010819
SFVmmu_K3T_KF026287
SFVmmu_A4W_KF026288
SFVhpi_SAM106
SFVggo
SFVggo-hu_CH101_KT211256
SFVggo-hu_H7Gab42_KT211261
SFVggo-hu_Bad551_KT211248
SFVggo-hu_Bak228_KT211246
SFVggo-hu__BAK74_JQ867464
SFVggo-hu_Sabak36_KT211249
SFVpvr_SFV7_KT211269
SFVptr_JudWd_KT211266
SFVptr-hu_H3Gab56_KT211267
SFVptr-hu_Bad316_KT211262
SFVptr-hu_H4Gab59_KT211268
SFVptr-hu_BAD327_JQ867463
SFVptr_hu_AG15_JQ867462
SFVptr_133Wd_KT211265
SFVpsc_hu
SFVpvr
SFVptr-hu_Pyl106_KT211263
SFVptr_Cam15_KT211264
SFVggo-hu_H2Gab54KT211257
SFVggo-hu_H5Gab27_KT211258
SFVggo-hu_Bad463_KT211253
SFVggo_Cam7_KT211250
SFVggo-hu_Bak82_KT211247
SFVggo-hu_Bak242_KT211254
SFVggo-hu_Bad456_KT211251
SFVggo-hu_H12Gab69_KT211260
SFVggo-hu_H6Gab51_KT211259
SFVggo-hu_Bad348_KT211252
SFVggo-hu_Bak46_KT211255
SFVggo-hu_BAD468_JQ867465
EFVeca
BFVbta
0.97
0.9
0.99
0.85
0.84
0.94
0.98
0.96
0.86
0.92
0.93
0.89
0.78
0.87
1
0.2

References

1. Price, M. N.; Dehal, P. S.; Arkin, A. P., FastTree 2--approximately maximum-likelihood trees for large alignments. *PLoS One* **2010,** 5, (3), e9490.
2. Galvin, T. A.; Ahmed, I. A.; Shahabuddin, M.; Bryan, T.; Khan, A. S., Identification of recombination in the envelope gene of simian foamy virus serotype 2 isolated from Macaca cyclopis. *J Virol* **2013,** 87, (15), 8792-7.
3. Richard, L.; Rua, R.; Betsem, E.; Mouinga-Ondeme, A.; Kazanji, M.; Leroy, E.; Njouom, R.; Buseyne, F.; Afonso, P. V.; Gessain, A., Cocirculation of Two env Molecular Variants, of Possible Recombinant Origin, in Gorilla and Chimpanzee Simian Foamy Virus Strains from Central Africa. *J Virol* **2015,** 89, (24), 12480- 91.
